# Pleurotus Mushrooms in Nutrition and Health: Clinical and Preclinical Insights for Nutraceutical Development

**DOI:** 10.1111/1541-4337.70279

**Published:** 2025-09-03

**Authors:** Patrícia Lima Araújo, Ediana da Silva Araújo, Erika Mayra de Almeida Barreto, José Luiz de Brito Alves, Kamylla Mylena Souza, Micaelle Oliveira de Luna Freire, Rayane Maria Pessoa de Souza, Fillipe de Oliveira Pereira

**Affiliations:** ^1^ Postgraduate Program in Nutritional Sciences Federal University of Paraíba João Pessoa Brazil; ^2^ Fungi Research Group, Academic Unit of Health, Education and Health Center Federal University of Campina Grande Cuité Brazil; ^3^ Department of Nutrition Federal University of Paraíba João Pessoa Brazil; ^4^ Multicentric Postgraduate Program in Physiological Sciences Federal University of Paraíba João Pessoa Brazil

## Abstract

*Pleurotus* mushrooms are fungi widely consumed due to their high nutritional value and potential applications as nutraceuticals. Their sustainable cultivation and rich composition of bioactive compounds provide significant health benefits. This review examines the scientific evidence regarding the safety, efficacy, and nutraceutical potential of *Pleurotus* species, focusing on their effects on various human diseases. The review incorporates findings from preclinical, clinical studies, and nutraceutical formulations related to innovative *Pleurotus*‐based products. Preclinical studies have shown that *Pleurotus* species can reduce inflammatory markers, modulate gut microbiota, and improve lipid and glucose metabolism. As a result, these mushrooms exhibit potential hypoglycemic, anti‐obesity, hepatoprotective, neuroprotective, anti‐atherogenic, and anticancer properties, along with possible benefits for preventing Alzheimer's disease. Clinical trials suggest that consuming *Pleurotus* has a positive effect on metabolic parameters in healthy individuals and patients with chronic conditions. However, the variability among studies and the absence of standardized nutraceutical formulations hinder definitive conclusions about their therapeutic efficacy. Despite the promising potential of *Pleurotus* mushrooms in the nutraceutical sector, future research should focus on developing standardized formulations, optimizing bioavailability, expanding clinical trials, exploring the diversity of native species, and uncovering the underlying mechanisms of action to establish their practical application as nutraceuticals.

## 1 Introduction

The consumption of mushrooms as food dates back to prehistoric times. Historically, mushrooms have been consumed as delicacies and utilized in medicinal practices and religious ceremonies by early civilizations, including the Chinese, Egyptians, Greeks, Indians, Latin Americans, and Romans (D.‐W. Li et al. 2016; O'Regan et al. 2016). Mushrooms are currently recognized as valuable sources of nutrients and bioactive compounds, exhibiting potential therapeutic effects for various human diseases (Shirur et al. 2021).

Edible mushrooms are consumed worldwide, although the species and quantities vary across regions. Approximately 2006 mushroom species are considered safe for human consumption (H. Li et al. 2021). However, only a fraction of these species is commercially cultivated. Among them, *Pleurotus* species, commonly known as oyster mushrooms, are widely recognized not only for their texture and flavor but also for their nutritional and functional properties. The group, which includes approximately 40 species, belongs to the family Agaricaceae within the class Basidiomycetes (Carvalho et al. 2024).

*Pleurotus* mushrooms account for approximately 25% of the global cultivated mushroom market, underscoring their significant contribution to the mushroom industry (Raman et al. 2021). The global edible mushroom market exceeds 34 billion tons annually, with *Pleurotus* species accounting for nearly 19% of this production. China leads the industry, producing approximately 87% of the world's *Pleurotus*, while other Asian countries contribute 12%, and Europe and the Americas account for only 1% (Juárez‐Hernández et al. 2023; Royse et al. 2017).

One key advantage of *Pleurotus* species is their cost‐effective and environmentally friendly cultivation technology. They can be grown on various lignocellulosic substrates, including agricultural residues and industrial by‐products, making them a sustainable food source (Albertó 2017; Dhar 2017). Examples of alternative substrates successfully used in *Pleurotus* cultivation include eucalyptus bark (Viriato et al. 2022), rice and corn straw (Zárate‐Salazar et al. 2020), banana leaves (Medeiros et al. 2024), and even urban waste, such as coffee grounds (Carrasco‐Cabrera et al. 2019). These features align *Pleurotus* mushrooms with the Sustainable Development Goals outlined in the 2030 Agenda (Grosso et al. 2020).

Beyond their sustainability, *Pleurotus* mushrooms are highly valued for their nutritional composition, which includes high levels of protein, dietary fiber, essential amino acids, vitamins (B and D), and minerals (Fe, Zn, Cu, Se) (Carrasco‐González et al. 2017; El‐Ramady et al. 2022). Additionally, they contain key bioactive compounds, including phenolic acids, flavonoids, terpenoids, ergosterol, and polyunsaturated fatty acids (ω‐3 and β‐glucans), which contribute to prebiotic and antioxidant activities (Andrade et al. 2024; Medeiros et al. 2024).

Among the numerous pharmacological properties of *Pleurotus*, two are highlighted due to their direct implications for human health: antioxidant activity (Ferreira et al. 2009) and prebiotic effects (Morales et al. 2021). Their β‐glucans and phenolic compounds can positively modulate gut microbiota, promoting intestinal health and immune function (Andrade et al. 2024). Recent findings suggest that gut microbiota plays a crucial role in host metabolic health, with notable immunomodulatory (Panda and Luyten 2022), antitumor (Elhusseiny et al. 2021), and cardiometabolic benefits (Dicks and Ellinger 2020).

*Pleurotus* mushrooms have shown a broad spectrum of potential therapeutic applications, as demonstrated by in vitro studies. However, the lack of animal models and human clinical trials remains a significant limitation. The presence of various bioactive compounds in these species has led to their use in the development of innovative food products. However, there is still a long way to go in establishing their effectiveness as nutraceuticals beyond their traditional roles as food or dietary supplements.

The rising cost of healthcare has fueled a growing demand for nutraceutical products across various sectors of society. Consequently, consumers increasingly turn to mushroom‐based nutraceuticals and dietary supplements to address nutrient deficiencies and incorporate bioactive compounds that may support overall health (Boccia and Punzo 2020). Moreover, many nutraceuticals and dietary supplements enter the market without comprehensive documentation on their quality, safety, and efficacy, and they are widely available without a prescription (Takefuji 2024).

Currently, consumers are increasingly interested in bioactive foods that offer health benefits and can help reduce the risk of diseases. Nonetheless, the lack of regulation surrounding these products raises concerns about their uncontrolled consumption. Reliable information on effective dosages, bioactive compound concentrations, and safety remains limited. This challenge is further complicated by varying regulations across countries and the inconsistent use of the term “nutraceutical” in scientific literature (Fernandes et al. 2024). Therefore, it is crucial to clearly define the origins of natural products, such as *Pleurotus* mushrooms; identify their bioactive compounds; and establish their safety and efficacy profiles, whether they are consumed as extracts or whole mushrooms.

While the interest in *Pleurotus* for functional foods and nutraceutical applications is increasing, systematic studies compiling preclinical and clinical trials remain inconclusive. In light of the emerging literature, this review compiles and analyzes relevant scientific findings on *Pleurotus* mushrooms as high‐value raw materials for nutraceutical applications, addressing this gap. Specifically, this study discusses their technological development, safety profile, preclinical and clinical efficacy, possible mechanisms of action, and potential health benefits. By providing this comprehensive perspective, we aim to support both the scientific community and consumers in understanding the role of *Pleurotus* mushrooms in human nutrition and well‐being, as well as the potential for nutraceutical development.

## 2 Material and Methods

We conducted this review in two steps: (1) a systematic literature search and (2) a narrative review to answer the following general question: What is the current evidence on the safety and efficacy of dietary supplements or nutraceuticals produced with *Pleurotus* spp.?

For the systematic search (1), we used the following databases: Embase, PubMed, Web of Science, and Scopus. The search strategy was adapted for each database using the keywords “*Pleurotus* AND nutraceutical OR dietary supplement OR functional food.” We considered original articles published in any language up to the 2000s as data sources. We applied the following inclusion criteria: nutraceuticals focused on human health, technological development of nutraceuticals, preclinical studies using animal models, or clinical studies involving humans. We excluded review articles, book chapters, articles on non‐*Pleurotus* species, studies focused on veterinary use, studies with solely chemical analyses, studies concerning mushroom cultivation or consumption, and studies involving isolated compounds. Duplicates were removed, and two independent reviewers performed a preliminary screening with the assistance of the Rayyan tool. A third reviewer was consulted in case of a tie. In this phase, articles that did not meet the selection criteria were excluded. Next, we conducted a full‐text review of potentially eligible articles to extract the data used in this article.

Finally, we conducted a narrative review (3) to gather current information on the nutraceutical formulations, processing, safety, and efficacy aspects of edible *Pleurotus* mushrooms, the pharmacological mechanisms involved in the therapeutic activities observed in the systematic literature search. In this context, we discussed and summarized the research findings, identifying gaps. For this review, we considered articles published in peer‐reviewed scientific literature databases, including Web of Science, Medline/PubMed, Embase, and Scopus.

**FIGURE 1:**
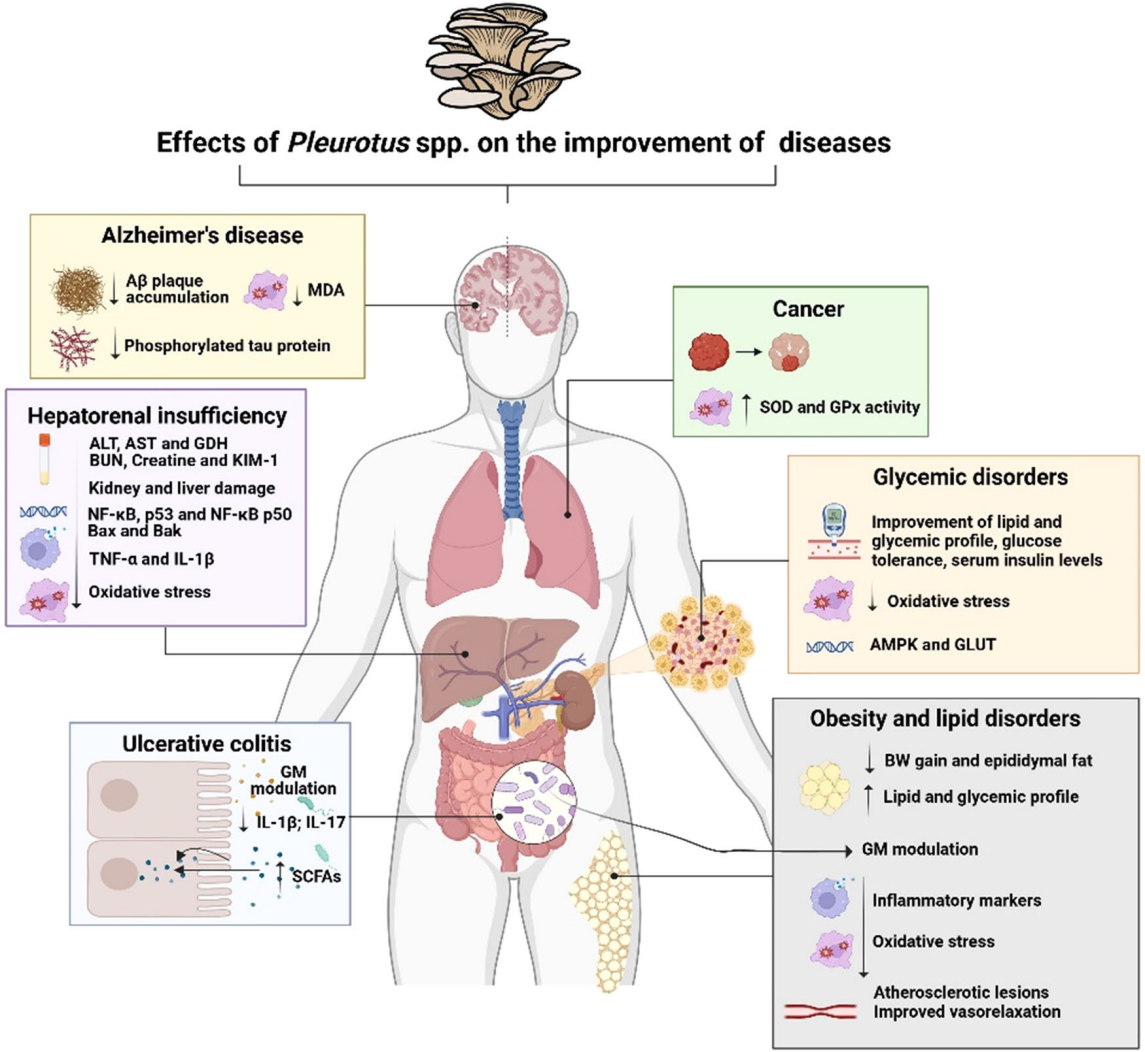
Mechanisms related to the effects of mushrooms in improving various diseases addressed in clinical and preclinical studies. Treatment with mushrooms has been found to improve intrinsic parameters of specific conditions, such as memory and learning ability in Alzheimer's disease, gastrointestinal symptoms in ulcerative colitis, improving liver and kidney function in hepatorenal failure, and reducing the number of tumor nodules in lung cancer. The studies indicate that mushrooms provide similar health benefits across clinical conditions. Their potential for promoting health is attributed to their anti‐inflammatory and antioxidant properties, improvement of lipid and glycemic profiles, and modulation of the intestinal microbiota. ALT: alanine aminotransferase; AMPK: AMP‐activated protein kinase; AST: aspartate aminotransferase; BUN: blood urea nitrogen; BW: body weight; GDH: glutamate dehydrogenase; GLUT4: glucose transporter 4; GM: gut microbiota; GPx: glutathione peroxidase; MDA: malondialdehyde; NF‐κB: nuclear factor kappa B; SCFAs: short chain fatty acids; SOD: superoxide dismutase.

To achieve the goals of this study, we organized our findings into four main topics. The first topic addresses safety concerns related to dietary consumption and preclinical toxicity studies of *Pleurotus* spp. The second topic provided an overview of the main functional aspects of *Pleurotus* spp. based on preclinical studies. The third topic reviews clinical evidence supporting the use of *Pleurotus* spp. for improving various health conditions. Finally, the fourth topic examines the development of innovative food products that utilize *Pleurotus* mushrooms. The key findings on how mushrooms affect various diseases in clinical and preclinical studies are illustrated in Figure 1.

## 3 Safety Aspects of *Pleurotus* Mushrooms

Edible mushrooms are valued for their nutritional and health benefits, but their safety is not guaranteed for all species. Some species contain toxic compounds that can cause allergic and hypersensitivity reactions (Venturella et al. 2021). The safety of *Pleurotus* mushrooms for human consumption has been verified by dietary consumption data in traditional and modern communities, in vitro and in vivo toxicity studies with mushroom products, chemical composition assessments to detected the presence of toxins or toxic elements acquired by mushrooms during cultivation or from the environment, and some case reports. This robust approach provides reliable information about the safety of *Pleurotus* mushrooms for human consumption.

In traditional food systems, populations consume *Pleurotus* mushrooms, especially in China and Mexico, two major mycophilic centers worldwide (Adedokun et al. 2022; Pérez‐Moreno et al. 2021). There are reports of *Pleurotus* consumption in Latin America, such as in Colombia (Cuesta and Castro‐Ríos 2017). The Wixarika and mestizo communities of Mexico report that they prefer eating mushrooms to other foods, including meat, especially *P. djamor*. They prepare the ground with fallen branches and trunks of the ochote tree (*Ipomea intrapilosa*) to allow them to grow (Haro‐Luna, Ruan‐Soto, and Guzmán‐Dávalos 2019; Pérez‐Moreno et al. 2020). *Pleurotus albidus* and *P. djamor* are the main species of mushrooms consumed by Yanomami in Brazil. They usually grow on tree trunks in areas where cassava cultivation is practiced (Sanuma et al. 2016). The *Pleurotus* genus includes edible species found in Brazil, which serve as a new food resource, according to a survey of species occurring in Brazil. A recently published list of edible mushrooms with confirmed edibility and occurrence in Brazil includes several species of *Pleurotus*, such as *P. albidus, P. djamor, P. fuscosquamulosus, P. pulmonarius*, and *P. Rickie* (Carvalho et al. 2024; Drewinski et al. 2024).

The consumption of *Pleurotus* is well‐established in modern food systems, indicating that eating the whole mushroom as food can be generally safe. The genus *Pleurotus* is among the most consumed in the world, but the quantities and species consumed of edible mushrooms may vary (Wan Mahari et al. 2020). Recently, some researchers proposed a system for categorizing mushroom species and assigning a final edibility status to clarify current knowledge on mushroom edibility. They found that at least 2006 species of mushrooms are considered safe for human consumption, and 183 species require some pretreatment before safe consumption (H. Li et al. 2021b). Thirty‐two of these mushrooms belong to the genus *Pleurotus*, including *P. citrinopileatus, P. djamor, P. eryngii*, and *P. ostreatus*. However, *P. ostreatus* is the most popular and consumed mushroom (Carrasco‐González et al. 2017).

Many *Pleurotus* species are cultivated commercially for use as food, dietary supplements, or food additives. However, the safe consumption of *Pleurotus* mushrooms, whether as whole food or in processed form, does not necessarily ensure their safety in nutraceutical formulations. This discrepancy is due to the various processing methods applied to the mushrooms, including drying, dehydration, and the use of different solvents to produce extracts. Certain toxins are often present in traditionally consumed mushroom species, such as *P. ostreatus*. One example is orellanine, a natural toxin that can produce oxygen radicals and inhibit protein synthesis through its metabolite. However, when the mushroom is processed, orellanine is deoxidized into orelin, which is non‐toxic (Nieminen and Mustonen 2020). Moreover, mushrooms may also absorb toxic components from the substrate used for cultivation, and there are complex interactions both among the mushrooms and between the products derived from them and the excipients included in pharmaceutical forms (Reis et al. 2017). Therefore, it is crucial to investigate the chemical profile and safety of these mushroom products under various processing conditions. This approach must be undertaken to provide a relative guarantee of safety before administering nutraceuticals.

Our review identified six preclinical studies using rodents as experimental models and one clinical study with healthy humans that evaluated the toxicity of *P. cystidiosus*, *P. florida*, *P. ostreatus*, and *P. tuber‐regium*, employing various extract types and mushroom forms and administered orally or inserted in the diet (Table 1).

**TABLE 1:** Preclinical and clinical evidence on the safety of *Pleurotus* mushrooms.

Species	Mushroom acquisition	Country	Toxicity test	Drug forms	Target population	Dose	Intervention time	Main results	Reference
*Pleurotus cystidiosus*	Cultivated, substrate not specified	Sri Lanka	Acute toxicity	Freeze‐dried powder	Wistar rats	500 mg/kg via oral gavage	6 weeks	No significant changes in behavior, and food and water consumption was constant. No significant differences in serum levels of ALT, AST, γ GT, creatinine, and hemoglobin.	(Jayasuriya et al. 2015)
*Pleurotus cystidiosus*	Cultivated, substrate not specified	Sri Lanka	Acute toxicity	Freeze‐dried powder	Healthy human	50 mg/kg orally	2 weeks	↓Fasting and postprandial serum glucose levels. No significant differences in serum levels of ALT, AST, γ GT, creatinine, and ALP.	(Jayasuriya et al. 2015)
*Pleurotus florida*	Cultivated, substrate not specified	India	Acute toxicity	95% acetone and 95% methanol	Female albino Wistar rats	2000 mg/kg via oral gavage	2 weeks	No changes in clinical signs, pathological abnormalities, and mortality.	(Karempudi et al. 2024)
*Pleurotus ostreatus*	Cultivated in pasteurized coffee pulp as substrate	Cuba	Acute toxicity	Aqueous extract	Male and female BALB/c mice	2000 mg/kg via oral gavage	2 weeks	No changes in health or behavior; No organ damage.	(Lebeque et al. 2018)
*Pleurotus ostreatus*	Cultivated	Finland	Acute toxicity	Powder	Female NIH/S mice	3, 6, or 9 g in diet	5 days	↓ Food intake; ↑ Water consumption; ↑ Plasma ALT activities; The potential toxic effects emerged in the present study after 5 days.	(Nieminen et al. 2009)
*Pleurotus ostreatus*	Cultivated, substrate not specified	Sri Lanka	Acute toxicity	Freeze‐dried powder	Wistar rats	500 mg/kg via oral gavage	6 weeks	No significant changes in behavior, and food and water consumption was constant. No significant differences in serum levels of ALT, AST, γ GT, creatinine, and hemoglobin.	(Jayasuriya et al. 2015)
*Pleurotus ostreatus*	Cultivated, substrate not specified	Sri Lanka	Acute toxicity	Freeze‐dried powder	Healthy human	50 mg/kg via oral gavage	2 weeks	↓Fasting and postprandial serum glucose levels. No significant differences in serum levels of ALT, AST, γ GT, creatinine, and ALP.	(Jayasuriya et al. 2015)
*Pleurotus ostreatus*	Wild	India	Acute toxicity	Powder	Female Sprague Dawley rats	5000 mg/kg via oral gavage 250, 500, 750, and 1000 mg/kg via oral gavage	3 days 28 days	No mortality and changes in the general behavior of rats were observed for 28 days of treatment.	(Deepalakshmi and Mirunalini 2014)
*Pleurotus tuber regium*	Wild	Nigeria	Metabolic and hematological toxicity induced by CCl_4_	Powder	Male Sprague Dawley rats	50–5000 mg/kg in diet	13 weeks	Reversal of food and water distortion; ↑ Hematological and immunological indicators; ↑ Activity of SOD, CAT, and GPx; ↑ GSH levels; ↑ Levels of ascorbic acid and α‐tocopherol; ↓ MDA levels.	(Okolo et al. 2020)

Abbreviations: ALP: alkaline phosphatase; ALT: alanine aminotransferase; AST: aspartate aminotransferase; CAT: catalase; CCl_4_: carbon tetrachloride; GPx: glutathione peroxidase; HFD: high fat diet; MDA: malondialdehyde; SOD: superoxide dismutase; γ GT: gamma‐glutamyltransferase.

Acute toxicological assessments of an ethanolic extract (5% w/v) of dried *P. ostreatus* powder in female Sprague‐Dawley rats have demonstrated a high margin of safety, with no toxic clinical symptoms or histopathological changes observed (Deepalakshmi and Mirunalini 2014). Another study evaluated the acute toxicity of *P. ostreatus* powder at concentrations of 1.8%, 3.6%, or 5.4% in the diet of female NIH/S mice for 5 days. The results indicated that *P. ostreatus* decreased food intake but increased water intake in a dose‐dependent manner, resulting in an increase in plasma alanine aminotransferase (ALT) activity (Nieminen et al. 2009). The acute toxicity of the aqueous extract of *P. ostreatus* was evaluated by oral administration of a single dose of 2000 mg/kg in male and female BALB/c mice. The animals were monitored for 14 days, and no significant changes were observed in clinical signs. Furthermore, macroscopic and histopathologic evaluations did not reveal organ damage, suggesting that the mushroom's ingestion has safe properties (Lebeque et al. 2018).

Prolonged administration of *P. ostreatus* and *P. cystidiosus* was verified in Wistar rats and healthy humans. The animals received a daily dose of 500 mg/kg for 6 weeks, while the humans received 50 mg/kg body weight orally. The experiment showed no significant changes in the behavior of the animals, and food and water consumption remained constant. Additionally, there were no significant differences in serum levels of ALT, aspartate aminotransferase (AST), gamma‐glutamyltransferase (GT), creatinine, and hemoglobin in animals and humans (Jayasuriya et al. 2015).

A study evaluated the acute toxicity of 95% acetone and 95% methanol extracts of *P. florida* in Wistar rats at a dose of 2000 mg/kg administered by oral gavage. During the toxicity test, no deaths or significant changes in the physiological or behavioral aspects of the animals were recorded over a 2‐week observation period (Karempudi et al. 2024). It has also been shown that a dose of 500 mg/kg of *P. tuber‐regium* (33.3% in the diet for thirteen weeks) ameliorated hematological toxicity caused by carbon tetrachloride (CCl_4_) in Sprague‐Dawley rats by reversing distortions in food and water consumption; improving hematological and immunological indicators; increasing the activity of antioxidant enzymes superoxide dismutase (SOD), catalase (CAT), glutathione peroxidase (GSH‐Px); and increasing the content of glutathione, ascorbic acid, and α‐tocopherol, in addition to promoting a reduction in malondialdehyde content (Okolo et al. 2020).

Although the data suggest that the consumption or use of *Pleurotus* mushrooms is generally safe and low in toxicity, caution is advised when consuming wild mushrooms from polluted environments. A study on *P. pulmonarius* grown in oil‐contaminated soil revealed hydrocarbon levels exceeding the non‐cancer daily reference dose, rendering it unsafe for human consumption (Adedokun et al. 2022). Moreover, cultivated *Pleurotus* spp. have been analyzed for their diverse elemental content, revealing differences in accumulation patterns and no health risks associated with consumption. Commercially available *Pleurotus* mushrooms (*P. ostreatus*, *P. eryngii, P. djamor*, *P. citrinopileatus*, *P. florida*, and *P. pulmonarius*) collected from their producers between 2009 and 2015 do not induce health risks to consumers due to their multi‐element content (Siwulski et al. 2017). Similarly, *Pleurotus* species cultivated on various agro‐industrial waste substrates were found to be safe for consumption, with health indices indicating no significant risk (Karataş 2022). Commercially cultivated *Pleurotus* mushrooms are safe and nutritious, but environmental factors should be considered when foraging wild specimens.

When considering mushrooms, it is essential to recognize the differences between consuming them whole and using extracts to ensure their safety and effectiveness. Each form offers benefits and applications, depending on the desired outcome. Whole mushrooms usually offer nutritional benefits and are functional foods. In contrast, mushroom extracts provide concentrated doses of bioactive compounds for targeted health applications, depending on the extraction method and solvent used. However, they can also induce undesirable reactions (Reis et al. 2017).

Although *Pleurotus* mushrooms are edible, some species, such as *Lentinus concavus* and *Amanita rubescens*, are safe for human consumption only when they are cooked or properly prepared (H. Li et al. 2021). In a study, fresh and dried *P. ostreatus* and *P. djamor* were used to prepare extracts by different extraction methods (maceration, soxhlet) and solvents (different ethanol/water ratios). The various processing methods presented extracts with varying profiles of chemical and biological effects (Martínez‐Flores et al. 2021). This highlights the importance of food processing in altering the nutritional, toxic, and pharmacological properties of the mushrooms.

Then, when developing mushroom‐based nutraceuticals, it is crucial to evaluate the toxicological profile of any edible mushroom, whether in powder, processed, or extract form, before proceeding with development, clinical studies, or commercialization. These studies provide valuable information for establishing safety limits to protect consumers from the potential adverse effects at high doses. Determining safe doses is crucial for both dietary consumption and use in nutraceuticals to ensure that the nutritional benefits of mushrooms can be enjoyed without health risks. Conducting studies on the composition and toxicity of mushrooms is crucial for identifying safe species, preventing adverse reactions, and ensuring that supplements and derived products do not pose health risks.

## 4 Use of *Pleurotus* Mushrooms in Preclinical Studies

The pharmacological properties of various *Pleurotus* mushroom species have been investigated in relation to several clinical disorders, including obesity, diabetes mellitus, atherosclerosis, colitis, Alzheimer's disease, hepatorenal injury, urolithiasis, and lung tumors. The reviewed preclinical studies employed different *Pleurotus* species in powdered or lyophilized forms, either incorporated into the diet or administered as oral organic extracts (Table 2). However, variations in mushroom preparation methods resulted in significant differences in the observed outcomes. Despite these discrepancies, the findings consistently demonstrate the pharmacological potential of *Pleurotus* across diverse animal models, regardless of the administration form. The data summarized in Table 2 have been organized and discussed in different sections according to the specific clinical disorders addressed in each study.

**TABLE 2:** Preclinical evidence on the effects of *Pleurotus* mushrooms.

Species	Mushroom acquisition	Country	Drug forms	Experimental models	Dose	Intervention time	Main results	References
*Pleurotus citrinopileatus*	Cultivated, substrate not specified	China	Ethanolic extract 90%	HFD‐induced obesity C57BL/6J male mice	200 mg/kg (low dose) and 500 mg/kg (high dose) via oral gavage	12 weeks	↓ Body weight gain and accumulation of epididymal fat; ↓ Food consumption; ↓Fasting blood glucose and glucose intolerance; ↓ Serum TC, TG levels; ↑Serum HDL levels; ↓Serum AST and LDH levels; ↓Serum creatinine levels;	(Chi et al. 2017)
*Pleurotus citrinopileatus*	Cultivated, substrate not specified	China	Aqueous extract	HFD‐induced obesity male C57BL/6J mice	400 mg/kg (low dose) e 800 mg/kg (high dose) via oral gavage	12 weeks	↓ Body weight gain and accumulation of epididymal fat; ↓ Food consumption; ↓ Serum TC, TG LDL, and non‐esterified fatty acids levels; ↑ Serum HDL levels; ↓ Serum AST levels; ↓Serum creatinine levels; ↓ Fasting blood glucose and glucose intolerance.	(Sheng et al. 2019)
*Pleurotus cystidiosus*	Cultivated, substrate not specified	Sri Lanka	Freeze‐dried powder	Alloxan‐induced diabetic Wistar male rats	500 mg/kg via oral gavage	6 weeks	↓ Postprandial glucose levels and intestinal contents; ↓ Serum GSK levels; ↑ Postprandial serum insulin and GK levels.	(Jayasuriya et al. 2012)
*Pleurotus eryngii*	Cultivated, substrate not specified	Taiwan	Powder	Alzheimer's disease induced by intracerebroventricular injection of β‐amyloid in male C57Bl/6J mice.	43 mg (low dose) and 107.5 mg (high dose) in diet	6 weeks	Prevented brain weight loss; ↓ Serum LDL levels; ↓ Levels of phosphorylated tau protein, MDA, protein carbonyls, and Aβ plaque accumulation in the brain; Improved memory and learning skills.	(Liang et al. 2020)
*Pleurotus eryngii*	Not specified	China	Powder	Dextran‐sodium‐sulfate‐induced colitis Crj: CD‐1 (ICR) mice	1% (low dose) and 3% (high dose) in diet	7 weeks	Absence of bleeding in the stool and improvement in stool consistency; Prevent colon shortening; Improve intestinal epithelial damage; ↓ Levels IL‐1β, IL‐17 in colon; ↓ Immune cell infiltration in the colon; ↑ Cecal levels of acetate, propionate, butyrate, and valeric acid; ↑ Richness and diversity indices of the gut microbiota; ↑ Abundance of S24‐7, Odoribacteraceae, *Adlercreutzia, Akkermanisa, Lactobacillus, Anaerostipes*, *Allobaculum;* ↓ Abundance of Actinobacteria, Desulfovibrionaceae, Enterococcaceae, *Turicibacter, Dorea, rc4‐4, Bacteroides*, and *Prevotella*.	(Q. Hu et al. 2019)
*Pleurotus eryngii*	Cultivated, substrate not specified	Japan	Freeze‐dried powder	Atherosclerosis model with apoE −/− mice	3% in diet	10 weeks	↓ Serum TC levels; ↓ Atherosclerotic lesions	(Mori et al. 2008)
*Pleurotus florida*	Cultivated, substrate not specified	India	Acetone and methanol extract 95%	Streptozotocin‐induced hyperglycemia female Wistar rats	200 mg/kg (low dose) e 400 mg/kg (high dose) via oral gavage	4 weeks	↓ Fasting blood glucose; ↓ Serum TC, TG, VLDL, and LDL levels; ↑ Serum insulin levels; ↓ Serum creatinine and BUN levels; ↓ Serum MDA and LH levels; ↑ Activity CAT, SOD, and GPx in the liver; ↑ GSH vitamin C e E levels in the liver; ↓ Pancreas, liver, and kidney damage.	(Karempudi et al. 2024)
*Pleurotus pulmonarius*	Not specified	Malaysia	Aqueous extract 10%	HFD‐induced hypercholesterolemia in Wistar‐Kyoto rats	0.5 and 2 g/kg via oral gavage	45 and 75 days	↓ Serum TC levels; Improved Ach‐dependent relaxation in aortic rings.	(Mohd Yahaya et al. 2022)
*Pleurotus ostreatus*	Cultivated on corncobs and cottonseed hulls substrates supplemented with sodium selenite and zinc sulfate	China	Powder	Healthy Kunming mice and mice with urethane‐induced lung tumors	5% (antioxidant effects) and 2% (antitumor effects) in diet	6 weeks (antioxidant effects) and 12 weeks (antitumor effects)	↑ GPx activity in blood; ↑ SOD activity in erythrocytes and liver; ↓ Number of tumor nodules on the lung surface.	(Yan and Chang 2012)
*Pleurotus ostreatus*	Cultivated, substrate not specified	Sri Lanka	Freeze‐dried powder	Alloxan‐induced diabetic Wistar male rats	500 mg/kg via oral gavage	6 weeks	↓ Postprandial serum glucose levels; Improved glucose tolerance; ↓ Fasting blood glucose; ↓ HbA1c levels.	(Jayasuriya et al. 2015)
*Pleurotus ostreatus*	Not specified	Egypt	Powder	Acetaminophen‐induced hepato‐renal injury male Swiss albino mice	10% in diet	10 days	↓ Serum ALT, AST, and GDH levels; ↓ Serum creatinine and BUN levels; ↓ Urinary KIM‐1 levels; ↓ Kidney and liver damage; ↓ MDA levels in kidney and liver; ↑ Activity of SOD and GPx; ↑ GSH levels.	(Naguib et al. 2014)
*Pleurotus ostreatus*	Cultivated, substrate not specified	Egypt	Aqueous extract 2%	Ethylene glycol‐induced urolithiasis male Wistar rats	100 mg/kg via oral gavage	8 weeks	↓ Serum creatinine e BUN; ↑ Urinary creatinine and BUN levels; ↓ Serum, urinary, and renal oxalate; ↓ Kidney damage; ↓ Serum TNF‐α and IL‐1β levels; ↑ Bcl‐2 expression in kidney; ↓ NF‐κB, p53, NF‐κB p65, and NF‐κB p50 expression in kidney; Bax and Bak expression in kidney.	(Ahmed et al. 2020)
*Pleurotus ostreatus*	Cultivated in wheat straw (*Triticum* spp.)	Mexico	Powder	Obesity‐induced by HFD and sucrose (10%) male Wistar rats	10% in diet	6 months	Attenuated weight gain; ↓ Size of adipocytes in subcutaneous adipose tissue; ↓ FM; Preserved the FFM; ↓ Serum of TC, TG and LDL levels; ↑ Adiponectin mRNA levels; ↓ BiP e XBP‐1 mRNA levels; ↓ JNK and p‐JNK mRNA levels; ↓ TNF‐α mRNA levels.	(González‐Ibáñez et al. 2023)
*Pleurotus ostreatus*	Cultivated, substrate not specified	Sri Lanka	Freeze‐dried powder	Alloxan‐induced diabetes Wistar male rats	500 mg/kg via oral gavage	6 weeks	↓ Postprandial glucose levels and intestinal contents; ↓ Serum GSK levels; ↑ Postprandial serum insulin and GK levels.	(Jayasuriya et al. 2012)
*Pleurotus ostreatus*	Cultivated, substrate not specified	China	Powder	HFD‐induced obesity C57BL/6J male mice	5% (low dose) and 10% (high dose) in diet	6 weeks	↓ Body weight gain and accumulation of subcutaneous epididymal and inguinal white adipose tissue; ↓ Fasting blood glucose; Improvement of glucose intolerance and insulin sensitivity; ↓ Serum TC, TG, and LDL levels; ↑ Abundance of Firmicutes, Ruminococcaceae, Peptococcaceae, Leuconostocaceae, Eubacteriaceae, Mogibacteriaceae, *Oscillospira, Lactobacillus, Bifidobacterium, Anaerostipes, Anaerovorax, Anaerofustis, Ruminococcus, Coprococcus*, and *Bilophila* e *Bacillus;* ↓ Abundance of Proteobacteria, Bacteroidetes, Desulfovibrionaceae, Helicobacteraceae, Beijerinckiaceae, Prevotellaceae, Paraprevotellaceae, *Bacteroides, Roseburia, Acinetobacter, Agrobacterium, Microbacterium, Novosphingobium, Streptococcus, Prevotella, Sphingomonas, Macrococcus*, and *Lactococcus;* ↑ Lipid and carbohydrate metabolism pathways; ↓ Adipocytokine signaling pathways and steroid hormone biosynthesis.	(Y. Hu et al. 2022)
*Pleurotus tuberregium*	Wild	Nigeria	Powder	HFD‐induced hypercholesterolemia Wistar male rats	5% and 10% in diet	4 weeks	↓ Body weight; ↓ Serum activities of ALT, AST, ALP, LDH, and CK; ↓ Serum of TC, TG, and LDL levels; ↑ Serum HDL levels; ↓ Serum and hepatic AChE activity; ↓ HMG‐CoA reductase activity and mevalonate concentrations in serum and liver; ↓ MDA and ROS levels; ↑ SOD, CAT, and GPx activity and GSH levels in liver and heart tissues.	(Oyetayo et al. 2020)

Abbreviations: 6PGD: 6‐phosphogluconate dehydrogenase; Ach: acetylcholine; AChE: acetylcholinesterase; ALT: alanine aminotransferase; AST: aspartate aminotransferase; Bak: BCL2 antagonist/killer; Bax: B cell lymphoma 2‐associated X protein; BiP: binding immunoglobulin protein; Bcl‐2: B‐cell lymphoma protein 2; BUN: blood urea nitrogen; CA3: carbonic anhydrase 3; CAT: catalase; Ccl‐2: C‐C motif chemokine ligand 2; Ccl‐3: C‐C motif chemokine ligand 3; CDH17: cadherin‐17; CK: creatine kinase; CRP: C‐reactive protein; CP: ceruloplasmin; FFM: fat‐free mass; FM: fat mass; GPx: glutathione peroxidase; HbA1c: glycosylated hemoglobin; HDL: high density lipoprotein; HFD: high‐fat diet; HMG‐CoA: hydroxymethylglutaryl‐coenzyme A; Hp: haptoglobin; G6PD: glucose‐6‐phosphate dehydrogenase; ICAM‐1: intercellular adhesion molecule‐1; IL‐1β: interleukin‐1 beta; IL‐17: interleukin‐17; JNK: p‐JNK: phosphorylated‐JNK; GSH: glutathione; KIM‐1: kidney injury molecule 1; LDL: low density lipoprotein; LDH: lactic dehydrogenase; LH: lipid hydroperoxides; ME malic enzyme; MDA: malondialdehyde; NF‐κB: nuclear factor kappa‐B; RGN: regucalcin; ROS: reactive oxygen species; SOD: superoxide dismutase; TC: total cholesterol; TG: triglycerides; TNF‐α: tumor necrosis factor alpha; VLDL: very low density lipoprotein; XBP‐1: X‐box binding protein 1.

### 4.1 Obesity and Associated Lipid Disorders

Obesity is a growing clinical disorder worldwide, associated with chronic diseases and changes in inflammation and oxidative stress markers (Oladimeji and Adebo 2024). Researchers have been exploring new approaches to treat obesity, and functional foods such as mushrooms can be a promising strategy. In our review, we found two studies that administered different doses of ethanolic extract (200 mg/kg and 500 mg/kg) and aqueous extract (400 mg/kg and 800 mg/kg) of *P. citrinopileatus* to obese C57BL/6J male mice. The mushrooms reduced body weight gain and epididymal fat accumulation, decreased food consumption, lowered fasting blood glucose levels, improved glucose intolerance, reduced serum triglyceride and AST, and increased serum high‐density lipoprotein levels (Chi et al. 2017; Sheng et al. 2019). The anti‐obesity effects of *P. ostreatus* (5% and 10% powder in the diet for 6 weeks) have been demonstrated in high‐fat diet (HFD)‐induced obese Crj: CD‐1 (ICR) mice. This treatment resulted in reduced body weight gain and the accumulation of subcutaneous epididymal and inguinal white adipose tissue, as well as a decrease in fasting blood glucose, improved glucose intolerance and insulin sensitivity, and a reduction in serum cholesterol, triglyceride, and low‐density lipoprotein (LDL) levels (Y. Hu et al. 2022).

Another study also demonstrated that *P. ostreatus* (10% in the diet for 6 months) attenuated weight gain, reduced adipocyte size in subcutaneous adipose tissue, fat mass, preserved fat‐free mass, and decreased serum levels of cholesterol, triglycerides, and LDL‐c in Wistar rats fed an HFD diet. Such improvements were accompanied by changes in the expression of parameters involved in inflammation and endoplasmic reticulum stress in subcutaneous adipose tissue, including increased protein expression of adiponectin and decreased expression of JNK (c‐jun N‐terminal kinase), p‐JNK (phosphorylated‐JNK), and TNF‐α (González‐Ibáñez et al. 2023).

The gut microbiota has been identified as a recent contributor to metabolic disorders, including obesity. The treatment with *P. ostreatus* promoted the modulation of the intestinal microbiota composition. This was characterized by an increase in the relative abundance of *Oscillospira*, the *Lactobacillus group*, and *Bifidobacterium*, while a decrease was observed in the relative abundance of *Bacteroides* and *Roseburia* (Y. Hu et al. 2022). A recent meta‐analysis evaluated the gut microbiome composition in obese and non‐obese individuals, reporting that *Bacteroides* and *Roseburia* are significantly more abundant in obese individuals. On the other hand, *Bifidobacterium* is more common in non‐obese individuals (Pinart et al. 2021). *Oscillospira* has been reported to produce short‐chain fatty acids (SCFAs) and prevent fat accumulation (J. Yang et al. 2021). *Lactobacillus* and *Bifidobacterium* may regulate lipid and carbohydrate metabolism and ameliorate obesity (Kim et al. 2024; Lee et al. 2024; S. Sun et al. 2024). In this sense, the beneficial effects of *P. ostreatus* on obesity may be partially mediated by the modulation of the gut microbiota.

In addition, pathways involved in lipid metabolism, including ketone body synthesis and degradation, bile acid biosynthesis, and fatty acid metabolism, are implicated in the beneficial effects of *P. ostreatus* on obesity (Y. Hu et al. 2022). Elevated bile acids have been involved in the modulation of parameters associated with obesity by stimulating energy expenditure through the TGR5 signaling, a G protein‐coupled receptor for bile acids expressed in many human cells, and stimulating mitochondrial division through the ERK/DRP1 (extracellular signal‐regulated kinase/GTPase dynamin‐1‐like protein) pathway in adipocytes (Velazquez‐Villegas et al. 2018).

Furthermore, the SCFAs generated from the gut microbiota play crucial roles in host metabolism. SCFAs enhance the production and release of the hormone peptide YY through the G protein‐coupled receptor GPR41. This promotes satiety by slowing down gut movement and reducing stomach emptying. SCFAs also impact insulin signaling and the formation of fat cells by boosting the production and release of leptin via GPR41 and GPR43. Butyrate stimulates the oxidation of fatty acids by inhibiting histone deacetylases and PPARα (peroxisome proliferator‐activated receptor alpha). Propionate and butyrate can stimulate intestinal gluconeogenesis, which partly explains the anti‐obesity and anti‐diabetic effects of fiber‐rich foods, such as mushrooms (Anachad et al. 2023). Butyrate and propionate downregulate adipocytokine signaling pathways and steroid hormone biosynthesis. Studies have reported that cytokines such as TNF‐α (tumor necrosis factor‐alpha), resistin, and IL‐6 are positively associated with obesity and that steroid hormones play a critical role in the metabolism, accumulation, and distribution of adipose tissue (Kushwaha et al. 2022; S. Zhao et al. 2021).

*Pleurotus tuber‐regium*, when included at 5% and 10% in the diet, resulted in significant health benefits for rats with hypercholesterolemia induced by HFD (Oyetayo et al. 2020). The treatment decreased body weight and serum levels of various biomarkers, including ALT, AST, lactate dehydrogenase (LDH), alkaline phosphatase (ALP), creatine kinase (CK), cholesterol, triglycerides, and LDL. Additionally, the study demonstrated a reduction in serum and liver activity of acetylcholinesterase (AChE) and a decrease in hydroxymethylglutaryl coenzyme A (HMG‐CoA) reductase activity, as well as lower concentrations of mevalonate in both serum and liver tissue. There was also a notable reduction in malondialdehyde (MDA) and reactive oxygen species (ROS) levels. Conversely, the treatment resulted in increased activity of antioxidants such as SOD, CAT, and glutathione peroxidase (GSHPx) in both liver and heart tissues.

The hypocholesterolemic properties of mushrooms may play a significant role in preventing various clinical conditions, including atherosclerosis. Research has shown that incorporating 3% *P. eryngii* into the diet for 10 weeks reduced total cholesterol levels and atherosclerotic lesions in apo E knockout mice (Mori et al. 2008). Additionally, the effects of *Pleurotus* spp. on vascular activity have been demonstrated. Specifically, treatment with *P. pulmonarius* (at doses of 0.5  and 2.0 g/kg for 45 and 75 days, respectively) resulted in reduced total serum cholesterol levels and improved acetylcholine (Ach)‐dependent relaxation in aortic rings of hypercholesterolemic rats (Mohd Yahaya et al. 2022).

*Pleurotus* mushrooms possess significant hypocholesterolemic and antiatherogenic properties. These mushrooms contain bioactive compounds, including polyphenols, lovastatin, polysaccharides, and ergothioneine, which contribute to their cardiometabolic health benefits (Cerletti et al. 2021; Uffelman et al. 2023). Changes in lipid profiles are often observed in conditions such as obesity and atherosclerosis. Consequently, the hypolipidemic effects of mushrooms may offer a potential alternative for treating these health issues (Uffelman et al. 2023; Venturella et al. 2021).

β‐Glucans, a type of polysaccharide found in mushrooms, are associated with reduced lipid profiles through several mechanisms. These compounds form a gel on the intestinal mucosa, inhibiting the reabsorption of bile acids and cholesterol. This stimulates bile acid biosynthesis in the liver, resulting in hypocholesterolemic effects by promoting the utilization of cholesterol (Cerletti et al. 2021; Sima et al. 2018). Additionally, β‐glucans bind to lipids and cholesterol, decreasing intestinal absorption and increasing fecal excretion. Mushrooms also contain lovastatin and promote SCFA production, inhibiting HMG‐CoA and reducing hepatic cholesterol synthesis (Cerletti et al. 2021; Sima et al. 2018; Uffelman et al. 2023).

Dietary polyphenols found in mushrooms can modulate cholesterol metabolism and reduce the risk of atherosclerosis (Murillo and Fernandez 2017). These compounds can decrease cholesterol absorption and plasma lipid levels, potentially through interactions with bile acids that inhibit the micellar solubility of cholesterol (P. Sun et al. 2021). They have also been shown to have antioxidant properties that can reduce LDL oxidation and circulating cholesterol, incorporate it into LDL particles, and enhance systemic antioxidant activity (Ahmadi et al. 2022).

### 4.2 Glycemic Disorders

The effects of *Pleurotus* mushrooms on regulating glycemic levels offer a potential alternative for preventing and treating type 2 diabetes mellitus (T2DM). A study using *P. ostreatus* and *P. cystidiosus* (500 mg/kg) in Wistar rats showed significant reductions in both fasting and postprandial glucose levels, accompanied by improved glucose tolerance. In diabetic models induced by alloxan (40 mg/kg), treatment with these mushrooms for 6 weeks reduced glycosylated hemoglobin (HbA1c) and improved metabolic markers (Jayasuriya et al. 2015).

Further investigations showed that these mushrooms lowered serum glucose and intestinal glucose content, reduced glycogen synthase kinase (GSK) levels, and increased insulin and glucokinase (GK) activity (Jayasuriya et al. 2015). GK is an enzyme primarily expressed in the liver and pancreas that initiates glucose metabolism by converting glucose to glucose‐6‐phosphate. Its upregulation enhances glucose uptake and utilization. Meanwhile, GSK is a negative regulator of glycogen synthesis, and its inhibition promotes glycogen storage in liver and muscle tissues (Abu Aqel et al. 2024; Borozdina et al. 2024). Thus, the hypoglycemic effects of *Pleurotus* mushrooms may be attributed to multiple mechanisms, including the stimulation of insulin secretion, enhancement of GK activity, and inhibition of GSK, collectively improving glucose metabolism and offering nutraceutical potential for T2DM management.

A study demonstrated that Wistar rats with streptozotocin (STZ)‐induced hyperglycemia, treated with methanolic and acetone extracts of *P. florida* (200 and 400 mg/kg for 4 weeks), exhibited reduced blood glucose levels and an improved lipid profile, accompanied by increased serum insulin levels. Additionally, these treatments enhanced renal function and decreased biomarkers of oxidative stress, such as MDA and lipid hydroperoxides. The mushrooms also boosted the activity of antioxidant enzymes in liver tissues, including CAT, SOD, GSH‐Px, and peroxides. Furthermore, they increased glutathione and vitamins C and E, which are non‐enzymatic antioxidant components (Karempudi et al. 2024).

Chronic hyperglycemia leads to insulin resistance, oxidative stress, and inflammation, all of which contribute to the progression of T2DM (González et al. 2023). The bioactive compounds in mushrooms—especially β‐glucans—have demonstrated the ability to increase insulin sensitivity, promote glycogen storage, and modulate gene expression of GSK‐3β, glycogen synthase, and glucose transporter 4 (GLUT4), particularly in hepatic and muscle tissues (Lin et al. 2018; Shamim et al. 2023). Additionally, *Pleurotus* mushrooms may regulate AMP‐activated protein kinase (AMPK), a key enzyme that enhances GLUT4 expression and translocation to the cell membrane, thereby facilitating glucose uptake by muscle cells and reducing circulating glucose levels (Asrafuzzaman et al. 2018; Lima et al. 2022).

Other pathways involved in the hypoglycemic effects of mushrooms include the PI3K/AKT signaling pathway, which plays a role in insulin‐mediated glucose uptake, as well as the inhibition of α‐amylase and α‐glucosidase, enzymes that break down complex carbohydrates into simple sugars, such as glucose. *Pleurotus* species may also reduce intestinal glucose absorption, further supporting glycemic control (Aramabašić Jovanović et al. 2021; Shamim et al. 2023).

Notably, hyperglycemia enhances the production of ROS, particularly in mitochondria, thereby contributing to cellular damage in insulin‐producing tissues. *Pleurotus* mushrooms may support glycemic balance by mitigating oxidative stress, thus preserving pancreatic β‐cell integrity and supporting insulin production (Karempudi et al. 2024; Rajlic et al. 2023).

### 4.3 Hepatorenal Insufficiency

Studies indicate that *Pleurotus* mushrooms can protect against liver and kidney dysfunction. Oxidative stress plays a key role in the development and progression of damage to both organs. When there is excessive production of ROS and an imbalance in antioxidant defenses, it leads to cellular injury, inflammation, and organ dysfunction. These processes are interconnected—ROS can trigger inflammatory pathways, while inflammation can promote the generation of additional ROS, creating a cycle of tissue damage (Lai et al. 2024; Simões e Silva 2025). In this context, oxidative stress is a central mechanism underlying hepatorenal injury and the progression of chronic diseases. Therefore, modulating oxidative stress and its downstream effects with *Pleurotus* mushroom represents a promising approach to protect these organs and slow the progression of disease.

The antioxidant properties of *P. ostreatus* mushrooms are primarily attributed to their phenolic acids, flavonoids, tocopherols, ascorbic acid, carotenoids, amino acids, and polysaccharides. These compounds exhibit antioxidant effects by scavenging superoxide and free radicals, donating hydrogen atoms, chelating ROS‐producing metal ions (such as Fe^2^⁺), and boosting antioxidant enzyme activity and non‐enzymatic antioxidant levels. They also inhibit xanthine oxidase and lipoxygenase, which are responsible for driving ROS production (Mwangi et al. 2022; Podkowa et al. 2021). Additionally, ergothioneine, an amino acid derivative found in mushrooms, is vital in protecting mitochondrial components from oxidative damage related to ROS generation (Fu and Shen 2022; Lam‐Sidun et al. 2021). Mushroom consumption may also activate the Nrf2/ARE pathway, which induces the synthesis of antioxidants and increases sirtuin 1 (SIRT1) activity, a factor involved in the cellular repair of damage caused by ROS (Luo et al. 2023; Song et al. 2021).

In a study, Swiss albino mice with acetaminophen‐induced hepatorenal injury treated with *P. ostreatus* (10% of the diet for 10 days) showed improved liver function through reduced serum ALT, AST, and glutamate dehydrogenase (GDH) levels and enhanced renal function by lowering serum creatinine and urinary kidney injury molecule 1 (KIM‐1) levels. Additionally, treatment reduced oxidative damage, evidenced by restored renal and hepatic tissue structure and improvements in oxidative stress markers, including decreased MDA levels and increased antioxidant enzyme activity (SOD and GSH‐Px) (Naguib et al. 2014).

Urolithiasis is a renal disease that causes urinary stones due to calcifications in the kidney, with hyperoxaluria being one of the significant risk factors for this clinical disorder (Allam 2024). A study showed that *P. ostreatus* aqueous extract (100 mg/kg for 8 weeks) exhibited nephroprotective effects in rats with ethylene glycol‐induced urolithiasis by reducing serum creatinine and urea levels while increasing urinary concentrations of these biomarkers. These improvements were further indicated by decreased serum, urine, and renal oxalate levels, lower urine specific gravity, and enhanced renal tissue structure. Treatment with *P. ostreatus* also lowered serum levels of inflammatory cytokines TNF‐α and IL‐1β, increased the anti‐apoptotic marker Bcl‐2 in renal tissue, and reduced the expression of inflammatory mediators (NF‐κB, p53, NF‐κB p65, NF‐κB p50), and pro‐apoptotic markers Bax and Bak (Ahmed et al. 2020).

The NF‐κB signaling pathway is a classic proinflammatory pathway that regulates cytokines such as TNF‐α and IL‐1β, playing a crucial role in hyperoxaluric conditions and kidney stone development (Ming et al. 2022). Oxidative stress, driven by various pathways, heightens the risk of stone formation, and the inflammatory response associated with kidney stones intensifies oxidative stress in a self‐perpetuating cycle. These mechanisms suggest that *P. ostreatus* may exert anti‐inflammatory and antioxidant effects, potentially improving the pathogenesis of urolithiasis and offering therapeutic benefits for other inflammation‐related conditions.

### 4.4 Intestinal Diseases

Ulcerative colitis is a chronic inflammatory bowel disease that increases the risk for colectomy and colorectal cancer. Symptoms of this disease include weight loss, blood in the stool, diarrhea, bloating, and constipation. However, the etiology of ulcerative colitis remains unclear (Gros and Kaplan 2023). Research suggests that the development of colitis may be associated with alterations in the intestinal microbiota. In this context, using prebiotic compounds, such as mushrooms, could be an alternative approach to improve conditions related to this disease (Q. Hu et al. 2019; Ikeda et al. 2024).

In this review, we found that *P. eryngii*, when included in the diet at concentrations of 1% and 3% for 7 weeks, effectively reduced the disease activity index in Crj: CD‐1 (ICR) mice suffering from dextran sodium sulfate (DSS)‐induced colitis. The absence of fecal bleeding and improved stool consistency evidenced this. Additionally, the mushroom prevented colon shortening by 10% and 15% and helped alleviate DSS‐induced damage to the intestinal epithelial wall. Other significant findings included decreased IL‐1β and IL‐17 levels and reduced immune cell infiltration in the colonic mucosa. Furthermore, there was an increase in the cecal concentrations of acetate, propionate, butyrate, and valeric acid (Q. Hu et al. 2019).

The modulation of the intestinal microbiota increased both the richness and diversity indices and changes in bacterial composition. At the family level, there was a notable rise in the S24‐7 and Odoribacteraceae families. At the genus level, increases were observed in *Adlercreutzia*, *Akkermansia*, *Lactobacillus*, *Anaerostipes*, and *Allobaculum*. Additionally, the treatment harmed the phylum Actinobacteria, the families Desulfovibrionaceae and Enterococcaceae. It also negatively affected the genera *Turicibacter*, *Dorea*, rc4‐4, *Bacteroides*, and *Prevotella* (Q. Hu et al. 2019).

The role of gut bacteria in the development of ulcerative colitis remains a topic of controversy. *Akkermansia* has been shown to reduce inflammation (Caesar et al. 2015). In contrast, *Akkermansia muciniphila* has been demonstrated to degrade mucin in the intestine, leading to intestinal barrier compromise, inflammation, and colonic tissue damage (Seregin et al. 2017). Inflammatory bowel disease was also associated with the decreased relative abundance of Bacteroidetes, Firmicutes, Lachnospiraceae, Ruminococcaceae, Erysipelotrichales, Bactereroidales, Clostridia, *Lactobacillus*, and *Bifidobacterium*. It increased the relative abundance of Enterobacteriaceae, Pasteurellcaea, Veillonellaceae, Fusobacteriaceae, *Ruminococcus gnavus*, and Desulfovibrio (Jauregui‐Amezaga and Smet 2024; W. Yang et al. 2022).

Increased SCFA production is a crucial mechanism by which mushrooms may benefit inflammatory bowel disease. Notably, butyrate provides energy to colonocytes; enhances intestinal barrier integrity; and has anti‐inflammatory effects by promoting Treg cell differentiation, reducing inflammatory cytokines, and regulating pathways like TLR4/MyD88/NF‐κB (Q. Hu et al. 2019; Y. P. Silva et al. 2020). Additionally, a study found that ergothioneine from *Pleurotus* mushrooms lowered protein expression of TLR4, MyD88, and NF‐κB p65 (Pang et al. 2022). These findings suggest that the prebiotic effects of *Pleurotus* mushrooms may help improve intestinal dysbiosis and protect against inflammatory bowel disease.

### 4.5 Neurological Disorders

The neuroprotective effects of mushrooms have been investigated in neurodegenerative diseases, particularly Alzheimer's disease (AD) (Liang et al. 2020; Liuzzi et al. 2023; Tong et al. 2023). AD is characterized by atrophy of the cerebral cortex and synaptic degeneration resulting from neuronal death, leading to impairments in motor, psychological, and memory functions. Pathological changes in AD involve amyloid beta (Aβ) plaques, which disrupt calcium balance, alter membrane potential, and increase apoptosis and synaptic loss (J. Zhang et al. 2024). Tau proteins aid microtubule formation and synaptic signaling, but their hyperphosphorylation plays detrimental pathological functions, ultimately leading to neurodegeneration (Lehmann et al. 2024). Oxidative stress also contributes to AD pathogenesis by increasing Aβ production, tau hyperphosphorylation, and mitochondrial dysfunction (Dhapola et al. 2024).

Recent investigations have explored the potential effects of *Pleurotus* in treating AD (Liang et al. 2020; Y. Zhang et al. 2016). The research demonstrated that feeding mice with AD, induced by an intracerebroventricular injection of β‐amyloid (Aβ), a diet containing *P. eryngii* (43 and 107.5 mg over 6 weeks) prevented brain weight loss. Additionally, it helped reduce levels of phosphorylated tau protein, MDA, and protein carbonyls in brain tissue while decreasing the accumulation of Aβ plaques. Behavioral tests further indicated that the treatment improved memory and learning abilities in the male C57Bl/6J mice (Liang et al. 2020).

The mechanisms by which mushrooms may improve Alzheimer's‐related parameters include enhancing antioxidant enzyme activities (SOD, GSH‐Px, and CAT), reducing MDA levels, lowering Aβ accumulation and tau phosphorylation, inhibiting beta‐secretase and AChE activity, reducing amyloid precursor protein (APP) expression, and increasing phosphatase 2A expression (A. M. Silva et al. 2023). Beta‐secretases cleave APP to generate Aβ peptides, which form amyloid plaques and aggravate AD, making their inhibition a therapeutic target (A. M. Silva et al. 2023; Tong et al. 2023). Additionally, the inhibition of AChE, which increases acetylcholine in the synaptic cleft, can help restore cholinergic function compromised in AD, supporting its value as a treatment approach (A. M. Silva et al. 2023; J. Zhang et al. 2024).

Recent research has highlighted the potential of mushroom‐derived compounds in combating AD, with bioactive substances including polysaccharides, ergothioneine, erinacine, polyphenols, alkaloids, ergosterol, and melanin. These compounds work through various mechanisms, including antioxidant and anti‐inflammatory effects, inhibition of apoptosis, and stimulation of neurite outgrowth (Tong et al. 2023). Specific components inhibit acetylcholinesterase and β‐secretase, prevent the aggregation of amyloid beta and neurotoxicity, and reduce tau expression and aggregation (A. M. Silva et al. 2023). Additionally, these bioactives target AD‐related pathways, including oxidative stress, neuroinflammation, and mitochondrial dysfunction (Dhapola et al. 2024). Ergothioneine, abundant in *Pleurotus* species, exerts antioxidant and anti‐inflammatory effects via the organic cation transporter 1 on neuronal membranes, helping to prevent cognitive decline (Gao et al. 2025). Polyphenols also activate protective pathways, including SIRT1, AMPK, and NRF2, which aid in reducing oxidative stress, neuroinflammation, and the accumulation of amyloid‐beta and tau deposits (García‐Aguilar et al. 2021).

While promising, further research is necessary to fully elucidate the efficacy and safety of mushroom‐derived compounds in treating and preventing AD. Expanded studies are needed to investigate how mushrooms impact these pathways to improve neurodegenerative parameters associated with AD.

### 4.6 Cancer

Lung cancer is the most common cancer globally and is a leading cause of cancer‐related deaths in both men and women (Kenaan et al. 2024). A study has shown the antitumor and antioxidant effects of *P. ostreatus* enriched with selenium and zinc. When Kunming mice with urethane‐induced lung tumors were treated with selenium‐enriched and zinc‐enriched *P. ostreatus* (2% mushroom powder in their diet for 12 weeks), there was a significant reduction in the number of tumor nodules on the lung surface, along with a restoration of GSH‐Px activity (Yan and Chang 2012). These findings suggest that *P. ostreatus*, in combination with selenium and zinc, may help inhibit tumor growth. This antitumor activity could be attributed to the beneficial immunomodulatory and regulatory effects of GSH‐Px. This enzyme requires selenium and is expressed in somatic cells to protect them from oxidative damage. Furthermore, GSH‐Px deficiency has been linked to tumorigenesis, while its overexpression is associated with a decrease in the growth rate of tumor cells (Y. Zhao et al. 2022).

## 5 Use of *Pleurotus* Mushrooms in Clinical Studies

Clinical studies have also examined the therapeutic properties of mushrooms, but the evidence and range of clinical applications are more limited compared to preclinical studies. Many studies identified in the systematic search focus on compounds isolated from mushrooms, such as polysaccharides and beta‐glucan, rather than the mushrooms themselves, which is the goal of this review. In this section, we highlight studies that used mushrooms in various forms, including freeze‐dried powder, extracts, baked goods, and fresh mushrooms consumed orally or incorporated into the diet, as shown in Table 3.

**TABLE 3:** Clinical evidence on the effects of *Pleurotus* mushrooms.

Species	Mushroom acquisition	Country	Drug forms	Subjects	Dose	Intervention time	Main results	References
*Pleurotus cornucopiae*	Company purchase	Japan	100% water extract	Healthy individuals	80 mL per pouch	8 weeks	↑ IFN‐γ and IL‐12 ↑ natural killer cell activity	(Tanaka et al. 2016)
*Pleurotus cystidiosus*	Cultivated, unspecified substrate	Sri Lanka	Freeze‐dried powder	Individuals with type 2 diabetes	50 mg/kg orally	6 weeks	↓ Fasting and postprandial serum glucose levels; ↑ Postprandial insulin levels.	(Jayasuriya et al. 2015)
*Pleurotus eryngii*	Not specified	Greece	Baked	Individuals with obesity	45 g of mushrooms in the meals	1 day	↓ Postprandial glucose levels; Regulated appetite; ↓ Postprandial ghrelin levels.	(Kleftaki et al. 2022)
*Pleurotus ostreatus*	Cultivated in sawdust (*Alnus rubra*)	USA	Freeze‐dried powder	Individuals with HIV and antiretroviral treatment‐induced hyperlipidemia	15 mg/day	8 weeks	↓ Triglycerides ↓ Non‐HDL cholesterol	(Abrams et al. 2011)
*Pleurotus ostreatus*	Not specified	Mexico	Fresh	Individuals with obesity	1 kg per week. Recommended to consume four portions of 250 g during the week	3 months	↓ Visceral fat and serum levels of glucose, cholesterol, and triglycerides in women; ↓ Serum triglyceride and glucose levels in men; ↓ Systolic and diastolic blood pressure in men.	(González‐Bonilla et al. 2022)

The hypoglycemic effects of *P. ostreatus* and *P. cystidiosus* were demonstrated in individuals with T2DM (Jayasuriya et al. 2015). A single dose of mushrooms was administered early, followed by a glucose load. The mushrooms reduced postprandial serum glucose levels and increased serum insulin levels in T2DM patients. The hypoglycemic effects of mushrooms are attributed to multiple mechanisms, including the increase in glucokinase activity, which promotes insulin secretion and glucose utilization by peripheral tissues, and the inhibition of GSK, which promotes glycogen synthesis in the liver (Abu Aqel et al. 2024; Teli and Gajjar 2023).

One study found that including *P. ostreatus* (1 kg per week) in a healthy diet for 3 months significantly affected metabolic markers in both women and men. In women, adding *P. ostreatus* reduced visceral fat and lowered blood glucose, cholesterol, and triglyceride levels. In men, the treatment resulted in lower levels of triglycerides and glucose and led to a decrease in systolic and diastolic blood pressure (González‐Bonilla et al. 2022). In another study, *P. ostreatus* did not reduce non‐HDL cholesterol levels in HIV patients experiencing antiretroviral treatment‐induced hypercholesterolemia. The minor decreases in HDL and triglyceride levels were not significant enough to justify further investigation (Abrams et al. 2011).

These findings suggest that edible mushrooms can be used as an adjunctive therapy for conditions such as dyslipidemia, T2DM, and arterial hypertension. *Pleurotus ostreatus*, in particular, is rich in bioactive compounds, including lovastatin, which is a natural inhibitor of HMG‐CoA reductase—the key enzyme involved in cholesterol synthesis. It also contains beta‐glucans, which help reduce lipid absorption and have a prebiotic effect, as well as ergosterol, which inhibits the absorption of exogenous cholesterol and promotes its excretion. Furthermore, it shows potential antidiabetic properties (Cateni et al. 2022).

A randomized, controlled, crossover clinical trial investigated the postprandial effects of *P. eryngii* in subjects with obesity (Kleftaki et al. 2022). Subjects were randomized to receive meals after an overnight fast. The test meal consisted of 100 g of white bread, 40 g of yellow cheese, and 45 g of *P. eryngii* mushrooms. After 1 month of follow‐up, subjects consuming *P. eryngii* had reduced postprandial glucose levels and improved regulation of appetite and ghrelin levels. Ghrelin stimulates food intake, promotes adiposity, increases body weight, and elevates blood glucose. Consequently, products that alter plasma ghrelin levels can potentially contribute to obesity and diabetes (Gupta et al. 2021).

Finally, the extract of *P. cornucopiae* significantly increased levels of IFN‐γ and IL‐12, as well as natural killer cell activity, in individuals who consumed 80 mL daily for 8 weeks. Together, these findings suggest that *P. cornucopiae* may enhance the immune system by potentiating the Th1 phenotype through macrophage–IL‐12–IFN‐γ signaling pathway (Tanaka et al. 2016).

It is noteworthy that the use of *Pleurotus* mushrooms in clinical studies varies depending on their form of administration (fresh, powdered, processed, or extracts). Fresh mushrooms offer a comprehensive nutritional profile but are highly perishable due to their high respiration rates, enzymatic activity, and water content. Consequently, preservation and processing methods are crucial for extending shelf life and minimizing microbial contamination. However, these methods can modify the chemical composition and biological activity of mushrooms, potentially altering their health effects (Yadav and Negi 2021).

Powdered and processed mushrooms may exhibit different physiological impacts compared to fresh mushrooms, primarily due to changes in bioactive compound concentrations and nutritional composition. While certain processing methods, such as drying and UV radiation, can enhance the retention or bioavailability of compounds like vitamin D2, others—such as boiling and frying—may reduce antioxidant activity and nutrient content (Kaur et al. 2023; Roncero‐Ramos et al. 2017). Moreover, freezing has been shown to decrease the levels of certain amino acids in *P. ostreatus* (Jaworska et al. 2011). These variations underscore the importance of selecting appropriate processing techniques to preserve the mushrooms' functional properties.

Therefore, the type of mushroom preparation used in clinical studies—whether extract, fresh, powdered, or processed—can significantly influence the observed health effects. Standardizing preparation methods and evaluating the bioaccessibility, bioavailability, and bioactivity of mushroom‐derived products are critical steps to ensure consistent and reliable outcomes in nutraceutical applications.

Furthermore, the advantages of mushrooms need more research through larger, longer, and well‐controlled clinical studies. Current research faces limitations, including small sample sizes and short intervention periods. Robust, long‐term randomized controlled trials are crucial for deepening our understanding of mushroom‐based interventions. This is crucial for evidence‐based clinical practice and could pave the way for new therapeutic options to complement conventional treatments.

## 6 Innovative food products using *Pleurotus* mushrooms

*Pleurotus* mushrooms are widely recognized for their applications in functional foods and serve as a valuable source of innovative food products. These mushrooms can be commercialized as dietary supplements or nutraceuticals, contributing to their broad consumption and application in promoting health and wellness.

The terms “dietary supplements” and “nutraceuticals” are often used interchangeably due to the absence of clear regulatory definitions. Dietary supplements address specific nutrient deficiencies and support overall health, particularly in populations with increased nutritional needs. They typically contain vitamins, minerals, amino acids, fatty acids, and other bioactive compounds (Mishra et al. 2021; Pralhad Jadhav et al. 2024). In contrast, nutraceuticals are food‐derived products that provide health benefits beyond essential nutrition, potentially reducing the risk of chronic diseases (Alali et al. 2021). Nutraceuticals encompass whole foods, isolated natural extracts, and bioactive chemical compounds from plants, mushrooms, and other organisms. Although nutraceuticals are available in pharmaceutical forms, their health benefits are not as rigorously evaluated as those of dietary supplements, which are strictly regulated and marketed with defined health claims (Fernandes et al. 2024; Reis et al. 2017).

Regulatory frameworks for dietary supplements and nutraceuticals vary globally. In the United States, the Food and Drug Administration (FDA) does not require dietary supplement manufacturers to demonstrate the safety and efficacy of their products, although a history of safety is necessary. In contrast, the European Food Safety Authority (EFSA) enforces stringent regulations regarding health and disease risk reduction claims, requiring toxicological data submission (Fernandes et al. 2024). In Brazil, dietary supplements and foods with functional or health‐related claims are regulated under specific guidelines; however, the term “nutraceutical” is not officially recognized. Edible mushrooms marketed as dietary supplements in Brazil are classified as oral ingestion products available in pharmaceutical forms (capsules, sachets, etc.) intended to supplement the diet of healthy individuals with nutrients and bioactive substances (ANVISA 2018).

The global nutraceutical market has been expanding steadily, driven by growing consumer interest in natural and organic products. Mushroom‐based nutraceuticals are expected to show an increasingly significant role in this market. The global edible mushroom market, including *Pleurotus*, was valued at USD 5.08 billion in 2021 and is expected to grow to USD 6.43 billion by 2028 (Niego et al. 2021). This growth is primarily driven by the rising demand for healthcare products in developing economies and the increasing appreciation for natural‐origin products (Nori and Manikiran 2022).

These products include liquids, powders, capsules, tablets, creams, gels, bars, lozenges, and chewing gums (Ali et al. 2019). The pharmaceutical formulation of nutraceuticals plays a critical role in ensuring the optimal release of bioactive compounds. Advanced pharmaceutical technologies enable the controlled release of nutrients and bioactive compounds, thereby enhancing their bioavailability and effectiveness. Additionally, these technologies improve consumer acceptability by masking undesirable flavors, aromas, or other sensory attributes (Zabot et al. 2022).

Among the strategies examined in this review, encapsulation emerges as one of the most promising for *Pleurotus*‐based formulations. Encapsulation techniques allow whole cells, nutrients, or bioactive molecules to be enclosed within protective matrices, shielding bioactive compounds from adverse environmental conditions and enhancing their stability and bioavailability (Reque and Brandelli 2021).

Despite notable advances, the term “nutraceutical” in the literature is primarily associated with the pharmacological or bioactive properties of mushrooms rather than the development of formulated nutraceutical products. Notably, none of the clinical studies reviewed here utilized the technological development of nutraceuticals using *Pleurotus* mushrooms, highlighting a critical gap in the development of nutraceutical formulations.

To the best of our knowledge, there are a few patents relevant to *Pleurotus*‐containing products. The United States Patent Application US20120128711 describes a formulation that combines a phytonutrient with *P. ostreatus* and *P. eryngii* enriched with vitamin D, designed to prevent and manage inflammation, oxidative stress, and related medical conditions. The Chinese patent CN110326784A presents a nutraceutical formulation containing *P. ostreatus* powder, mycelial parts of other fungal species to support hepatic metabolism.

Our findings show that the number of innovative products related to *Pleurotus*‐based nutraceuticals remains limited. While bioactive compounds from *Pleurotus* spp. have been extensively studied for their potential health benefits, including antioxidant, anti‐inflammatory, and immunomodulatory effects, limited data exist on their stability and efficacy when incorporated into delivery systems such as capsules, tablets, or functional foods (Mayirnao et al. 2025). This gap highlights the urgent need for further research focused on the formulation of *Pleurotus*‐derived products into standardized, clinically validated nutraceuticals. Additionally, laboratory techniques and industrial processes must be refined to enable the large‐scale production of these products at commercially viable costs. Addressing these challenges will unlock the vast innovation potential of *Pleurotus* mushrooms, supporting the development of evidence‐based nutraceutical formulations and meeting the growing demand for natural, functional foods in global markets.

## 7 Conclusion

This review highlights that *Pleurotus* mushrooms have emerged as a promising resource for nutraceutical applications due to their rich nutritional profile, bioactive compounds, and sustainable cultivation. However, key challenges remain. One crucial issue is the need for standardized nutraceutical formulations and routes of administration, as these factors affect the chemical and pharmacological properties of the nutraceutical. To address this, the development of pharmaceutical formulations and digestion studies are essential to analyze the impact on the bioaccessibility and bioavailability of *Pleurotus* bioactive compounds. Additionally, while numerous in vitro and animal studies support their health benefits, randomized clinical trials must be expanded to validate their efficacy in diverse human disease models. Future research should also aim to elucidate the possible mechanisms of the action of *Pleurotus* mushrooms and their bioactive compounds, thereby improving the understanding of their beneficial and potential adverse effects. Finally, expanding investigations to include native *Pleurotus* species may lead to the discovery of novel bioactive compounds and a deeper understanding of fungal biodiversity in food systems. Addressing these gaps will optimize the rational utilization of *Pleurotus* mushrooms and expand their applications in disease prevention and health promotion.

## Author Contributions

**Patrícia Lima Araújo**: conceptualization, writing–original draft, investigation, formal analysis. **Ediana da Silva Araújo**: investigation, methodology, writing–original draft. **Erika Mayra de Almeida Barreto**: writing–original draft, methodology, investigation. **José Luiz de Brito Alves**: supervision, formal analysis, conceptualization, writing–review and editing. **Kamylla Mylena Souza**: investigation, methodology, writing–original draft. **Micaelle Oliveira de Luna Freire**: investigation, methodology, writing–original draft, writing–review and editing. **Rayane Maria Pessoa de Souza**: investigation, writing–original draft, methodology. **Fillipe de Oliveira Pereira**: conceptualization, investigation, supervision, project administration, writing–review and editing.

## Conflicts of Interest

The authors declare no conflicts of interest.

## Data Availability

All data are presented in the current study.
